# Gait Characteristics of Dynapenia, Sarcopenia, and Presarcopenia in Community-Dwelling Japanese Older Women: A Cross-Sectional Study

**DOI:** 10.3390/healthcare10101905

**Published:** 2022-09-28

**Authors:** Kohei Mori, Shin Murata, Akio Goda, Yuki Kikuchi, Kayoko Shiraiwa, Jun Horie, Hideki Nakano

**Affiliations:** 1 Graduate School of Health Sciences, Kyoto Tachibana University, Kyoto 607-8175, Japan; 2 Faculty of Allied Health Sciences, Kansai University of Welfare Sciences, Osaka 582-0026, Japan; 3 Faculty of Health Science, Kyoto Tachibana University, Kyoto 607-8175, Japan

**Keywords:** dynapenia, sarcopenia, gait, older, presarcopenia

## Abstract

Age-related decline in skeletal muscle mass and function are risk factors for reduced walking ability. This study aimed to understand the characteristic gait parameters of presarcopenia (low muscle mass only), dynapenia (low muscle function only), and sarcopenia (low muscle mass and function), which have differing skeletal muscle characteristics. Skeletal muscle mass, grip strength, and gait parameters (walking speed, cadence, step length, step width, gait angle, foot angle, stance time, swing time, and double stance time) were evaluated in 307 older Japanese women. Low muscle function was determined by grip strength and normal walking speed. Participants were assessed and divided into the normal (60.9%, *n* = 187), presarcopenia (25.7%, *n* = 79), dynapenia (5.2%, *n* = 16), and sarcopenia (8.1%, *n* = 25) groups. When compared to the normal group, the sarcopenia group had significantly slower walking speed and shorter step length (*p* < 0.05); the dynapenia group had significantly slower walking speed, smaller cadence, shorter step length, wider step width, and longer stance time (*p* < 0.05); and the presarcopenia group showed no differences. Skeletal muscle function may therefore be more strongly related to reduced walking function in older adults than body composition factors. The decrease in walking function was most pronounced in older women with dynapenia.

## 1. Introduction

In older people, walking ability is a predictor of reduced activities of daily living, fall risk, institutionalization, and mortality [1,2,3,4,5]; therefore, maintaining the ability to walk is of paramount importance to support the health of older adults. In particular, older women have shown a faster rate of decline in walking speed with age [6] and a higher prevalence of gait disorders than men [7]. Age-related decline in skeletal muscle mass and strength are risk factors for reduced walking ability [8], and efforts to prevent and reverse sarcopenia in older adults have attracted attention worldwide.

Sarcopenia is a concept proposed by Rosenberg in 1989 as a form of age-related skeletal muscle loss [9]. Subsequently, the European Working Group on Sarcopenia in Older People [10] proposed defining sarcopenia as a condition in which reduced muscle function (muscle strength or physical performance) is combined with age-related decline in skeletal muscle mass. The Asian Working Group for Sarcopenia (AWGS) [11,12] set cut-off values for skeletal muscle mass and function (grip strength and walking speed) for Asian people, and its diagnostic criteria are widely used in Japan. However, it has been pointed out that muscle weakness associated with aging progresses about three times faster than muscle mass declines; thus, loss of muscle mass can only partially explain the decline in strength [13]. Clark et al. [14,15] defined age-related muscle functional decline that is independent of skeletal muscle mass as dynapenia, proposing a concept that differs from sarcopenia. Sarcopenia and dynapenia, as determined by their respective definitions, are associated with adverse health outcomes such as falls [16,17], reduced activities of daily living [18], and increased risk of mortality [19,20].

Changes in gait parameters associated with age include decreased walking speed, reduced stride length, increased stride width, decreased cadence, and prolonged double stance time [21]. In previous research on the associations between muscle mass, strength, and walking ability, many studies have found that strength correlates positively with walking speed. Conversely, correlations between muscle mass and walking speed have not been observed [22,23,24], indicating that strength has a major impact on walking speed but muscle mass does not. However, there have also been reports of reduced muscle mass with reduced mobility [8,25], and it remains unclear the effects on temporal and spatial gait parameters when muscle mass loss and muscle function loss coexist, or when each is present alone. Further, the assessments of walking function used in these studies were limited to questionnaires and measuring walking speed. They did not examine changes in any other temporal or spatial parameters. Gait is an extremely complex motor task that can be described by parameters other than walking speed, such as cadence, step length, step width, swing, stance, and double support times. Previous studies have shown that the greater the number of parameters indicative of low gait function, the higher the cumulative risk of developing disability [26]. Therefore, multidimensional and quantitative gait analysis can be an important means of understanding the signs of disability development in the elderly.

The objective of the present study was to clarify the characteristic gait parameters of dynapenia (muscle function decline without reduced muscle mass), presarcopenia (reduced muscle mass without muscle function decline), and sarcopenia (reduced muscle mass and function), which have different skeletal muscle characteristics according to the AWGS criteria [12].

## 2. Materials and Methods

### 2.1. Participants

This was a cross-sectional study of older residents of Yasu, Shiga Prefecture, Japan, conducted from August 2017 to September 2019. A total of 410 participants were recruited through leaflets and posters distributed to people taking part in an annual health survey of the older population. If a participant took part in the survey multiple times between 2017 and 2019, the data from the first survey was used in the cross-sectional study.

**Figure 1:**
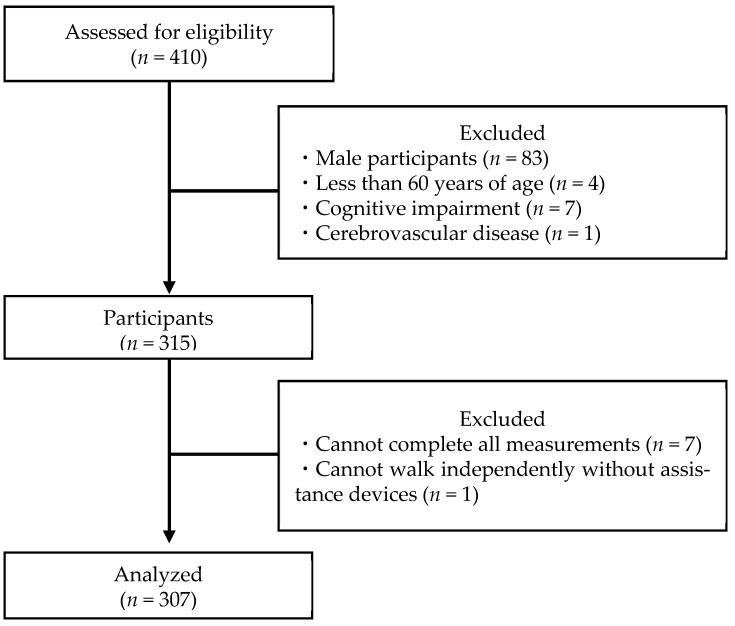
Participant selection flowchart.

After collecting data regarding a participant’s age, height, and weight, the participant completed a Mini-Mental State Examination (MMSE) to assess their cognitive functions [27,28]. The inclusion criteria for this study were (1) age ≥ 60 years and (2) absence of cognitive impairment (MMSE ≥ 24). The exclusion criteria were (1) male, (2) history of cerebrovascular disease, (3) severe orthopedic disease of the lower extremities, (4) unable to walk independently without walking aids, and (5) not having undergone all measurements. After excluding those who met the exclusion criteria, 307 participants formed the set for final analysis (Figure 1). The sample size was calculated using G * Power [29]. G power was set as follows: test family, F tests; statistical test, analysis of covariance (ANCOVA); effect size, 0.44; α error prob, 0.05; power (1-β error prob), 0.80; numerator, 3; number of groups, 4; and number of covariates, 2. The total sample size was calculated as 61.

The objective of this study and how personal information and the data that were collected would be handled were explained in writing to the participants, and their consent was obtained. This study complied with the Declaration of Helsinki and was conducted with the approval of the ethical review board of Kyoto Tachibana University (approval numbers 17-14 and 18-26). 

### 2.2. Measurements

#### 2.2.1. Skeletal Muscle Mass

Muscle mass was measured with a portable body composition analyzer (InBody 470, InBody Japan Inc., Tokyo, Japan) using the bioelectrical impedance method [30]. Skeletal muscle mass index (SMI) was calculated as the total of the skeletal muscle mass (kg) of the limbs divided by the square of the height (m^2^).

#### 2.2.2. Muscle Strength

Grip strength was used as the indicator of muscle strength. Grip strength was measured using a Smedley dynamometer (T.K.K. 5401, Takei Scientific Instruments Co., Ltd., Niigata, Japan) [31]. The participants were instructed to stand with their legs shoulder-width apart and their arms hanging naturally while they gripped the dynamometer with maximum effort. The left and right sides were each measured twice, and the mean of the maximum values was used as the representative value.

#### 2.2.3. Gait Parameters

**Figure 2:**
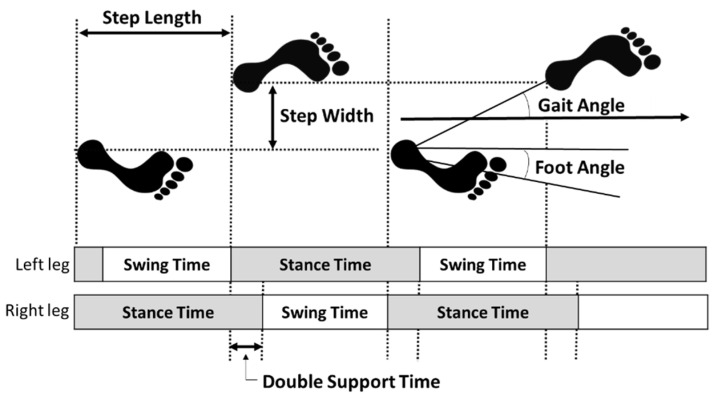
Gait parameters.

The gait parameters were measured with a WalkWay device (WalkWay MW-1000; Anima Co., Tokyo, Japan) [32,33]. This device calculates temporal and spatial gait parameters from the distribution of foot pressure and consisted of a sheet size of 2400 mm length × 800 mm width × 5 mm thickness, sensor spatial resolution of 10 mm × 10 mm, and 14,400 measurement points. The participants were asked to walk a total of 6.4 m (the gait section), which consisted of a 2 m acceleration section, 2.4 m of the Walkway device sheet (the measurement section), and then 2 m of deceleration. The data was measured at a sampling rate of 100 Hz. Participants were instructed to “walk at a regular speed”, barefoot, and two measurements at normal walking speed were performed [33]. The analysis was performed on the following calculated factors: walking speed, cadence, step length, step width, gait angle (angle between the straight line and direction of travel from one foot landing to the other), foot angle (angle between the toes and the direction of travel), stance time, swing time, and double stance time (Figure 2). The means of the left and right values were used for step length, gait angle, foot angle, stance time, and swing time.

### 2.3. Assessing Presarcopenia, Dynapenia, and Sarcopenia

**Figure 3:**
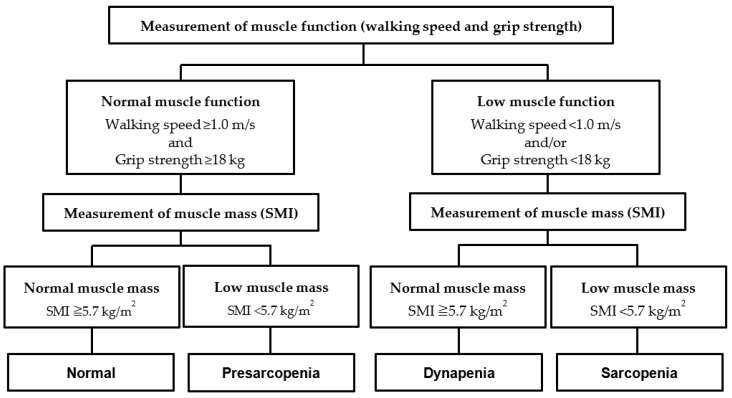
Assessment of normal, presarcopenia, dynapenia, and sarcopenia. SMI, skeletal muscle mass index.

We classified the participants into four groups—normal, presarcopenia, dynapenia, and sarcopenia—based on the results of the grip strength, normal walking speed, and SMI measurements, referencing a study by Yamada et al. [34] (Figure 3). The sarcopenia diagnostic criteria from AWGS 2019 [12] were used for the assessments, with muscle functional decline defined as a grip strength of <18 kg and/or normal walking speed of <1.0 m/s, and reduced muscle mass as SMI < 5.7 kg/m^2^. Therefore, participants in the normal group had normal muscle mass and function, those in the presarcopenia group had low muscle mass and normal muscle function, those in the dynapenia group had normal muscle mass and low muscle function, and those in the sarcopenia group had low muscle mass and muscle function.

### 2.4. Statistical Analysis

One-way analysis of variance was used to compare basic attributes (age, height, weight, and body mass index [BMI]) and indicators of sarcopenia (SMI, grip strength, and walking speed) between the normal, presarcopenia, sarcopenia, and dynapenia groups. Analysis of covariance was used to compare gait parameters between the four groups, with age and BMI as covariates. Items with significant differences underwent a multiple comparison test using the Bonferroni method. IBM SPSS Statistics for Windows Version 28.0 (IBM Japan, Ltd., Tokyo, Japan) was used for the analysis, and the significance level was 5%.

## 3. Results

Table 1 shows the basic attributes of all the groups. Of the 307 participants in the study, 60.9% (*n* = 187) were assessed as normal, and 25.7% (*n* = 79), 8.1% (*n* = 25), and 5.2% (*n* = 16) were assessed to have presarcopenia, sarcopenia, and dynapenia, respectively. There were significant differences in age, height, weight, and BMI (*p* < 0.001). The sarcopenia group was significantly older and shorter than the normal and presarcopenia groups (*p* < 0.01). In addition, the presarcopenia and sarcopenia groups had significantly lower body weight than the normal and dynapenia groups (*p* < 0.01). The BMI was significantly lower in the presarcopenia group than in the normal and dynapenia groups and was lower in the sarcopenia group than in the normal group (*p* < 0.001).

Table 2 shows the results of covariance analysis of the gait parameters in the four groups, with age and BMI as covariates. Regarding the gait parameters, walking speed was significantly slower and step length significantly shorter in the sarcopenia group than in the normal group (*p* < 0.05). Walking speed in the dynapenia group was significantly slower than in the other three groups, while step width and gait angle were larger (*p* < 0.05). Cadence was significantly smaller and step length shorter than the normal and presarcopenia groups (*p* < 0.05), while stance time was significantly longer than the normal group (*p* < 0.05). Notably, none of the gait parameters in the presarcopenia group differed significantly from those in the normal group.

## 4. Discussion

The objective of this study was to clarify the characteristic gait parameters of older women with presarcopenia, sarcopenia, and dynapenia, conditions that have different skeletal muscle characteristics. The results showed that women with sarcopenia and dynapenia had different gait parameter characteristics. Participants with sarcopenia had reduced walking speed due to decreased step length, whereas those with dynapenia had reduced step length as well as low cadence, causing the most pronounced decline in walking speed. In addition, participants with dynapenia had larger step width and gait angle than those in the other three groups, suggesting that they were compensating for an unsteady gait. In contrast, the gait parameters of participants with presarcopenia were no different from normal participants.

Walking speed is an effective and reliable indicator of physical function and health in older adults [35] and is defined by stride length and cadence. Doi et al. [26] reported that walking speed, stride length, cadence, and stride length variability were predictors of disability onset in a longitudinal study of 4121 older Japanese participants. The study calculated cut-off values for each gait parameter (walking speed: 1.10 m/s; cadence: 116.5 steps/min; stride length: 1.15 m; stride length variability: 2.86%) and reported that the more the number of parameters indicating low walking function, the higher the risk of disability onset. Another study with a similar design used death as an outcome [36] and reported that the more the number of parameters indicating low walking function, the higher the mortality risk. Although we did not examine stride length variability, in the sarcopenia group, only stride length was lower than the cut-off values, as published by Doi et al. [26]; in the dynapenia group, walking speed and stride length were lower, although cadence was close to the cut-off value. These results suggest that the dynapenia group is more prone to having a walking function that leads to adverse health outcomes than the sarcopenia group.

An increase in step width is a characteristic change in gait parameters in older people at risk of falling [37]. Kao et al. [17] investigated the risk of falling in older adults with dynapenia, presarcopenia, and sarcopenia. They reported that after adjusting for several covariates, the odds ratio for a fall event was highest in the dynapenia group (odds ratio: 3.11), followed by the sarcopenia (odds ratio: 2.80) and presarcopenia (odds ratio: 0.85) groups. In the present study, the dynapenia group had a significantly larger step width than the other three groups, which may indicate a compensatory action for an unstable gait. A wider step width may also indicate a high risk of falls in older people with dynapenia.

The presarcopenia group exhibited no differences in gait parameters compared to the normal group. Schaap et al. [38] conducted a meta-analysis of 50 articles and found that low muscle strength was associated with functional decline, whereas low muscle mass had no significant association. In addition, Hayashida et al. [24] found that in a cross-sectional study of older Japanese community-dwelling participants, muscle strength had a significant positive correlation with walking speed, while muscle mass did not. We confirmed these previous findings and showed that reduced skeletal muscle mass alone is not associated with a decrease in walking speed, and it is also not associated with any temporal or spatial parameters, including stride length, stride width, and double stance time.

The results of this study showed gait parameters indicating lower gait function in dynapenia and sarcopenia, where muscle function loss occurred, but not in presarcopenia, where only muscle mass loss was present. These results suggest that skeletal muscle functions, such as strength, are more strongly related to reduced gait function in older adults than are body composition factors, such as skeletal muscle mass.

This study had several limitations. First, while we showed that gait function in dynapenia is significantly lower than in sarcopenia, we did not demonstrate its mechanism. The onset of dynapenia is presumed to involve some kind of nervous system impairment (such as a decrease in cerebral cortex excitability or a decrease in the maximum firing frequency of motor units) or muscular system impairment (such as an increase in the amount of intramuscular fat infiltration or dysfunction of excitation-contraction coupling) besides reduced muscle mass [15]. Therefore, going forward it would be useful to examine this condition from neurological and physiological perspectives. Second, the participants were active older women who enthusiastically participate in community activities, which may limit the generalizability of the findings to other populations of older individuals. Further study of populations that include older men and frail older people is also needed. Third, the effect of chronic diseases possessed by the subjects has not been taken into account. It is undeniable that differences in the prevalence of chronic diseases in each group may affect the degree of gait dysfunction. Future studies should therefore take into account the influence of chronic diseases based on accurate diagnosis by medical institutions. In addition, future work will require comparisons among age-matched groups to strictly take into account the effect of age.

## 5. Conclusions

This study compared gait parameters in participants with dynapenia, sarcopenia, and presarcopenia and compared their measurements with participants whose measurements were assessed as normal. While participants in the sarcopenia group exhibited reduced walking speed and shorter step length, those in the dynapenia group exhibited these signs along with reduced cadence, increased step width, and longer stance time, which confirmed that the decline in walking function was pronounced in this group. In contrast, none of the parameters indicating reduced gait function changed in participants in the presarcopenia group. Our findings indicate that gait function is related to muscle function rather than muscle mass and that gait dysfunction is more pronounced in the elderly with dynapenia as well as sarcopenia.

## Figures and Tables

**Table 1:** Basic characteristics of the participants.

	Normal (*n* = 187)	Presarcopenia (*n* = 79)	Sarcopenia (*n* = 25)	Dynapenia (*n* = 16)	ANOVA	
Characteristic					F Value	*p* Value
Age (y)	73.0 ± 5.4	73.5 ± 5.6	77.8 ± 6.7 ^N,P^	75.8 ± 7.1	6.0	<0.001
Height (cm)	152.3 ± 5.0	151.3 ± 5.1	146.0 ± 4.8 ^N,P^	149.3 ± 7.0	12.0	<0.001
Weight (kg)	53.6 ± 7.0	45.4 ± 5.4 ^N,D^	44.5 ± 4.6 ^N,D^	51.0 ± 4.8	39.7	<0.001
BMI (kg/m^2^)	23.1 ± 2.9	19.8 ± 2.3 ^N,D^	20.9 ± 2.4 ^N^	22.9 ± 1.7	30.1	<0.001
SMI (kg/m^2^)	6.42 ± 0.58	5.34 ± 0.29 ^N,D^	5.19 ± 0.45 ^N,D^	6.04 ± 0.22 ^N^	111.8	<0.001
Grip strength (kg)	25.2 ± 3.4	22.7 ± 2.6 ^N^	17.3 ± 3.5 ^N,P^	19.0 ± 3.7 ^N,P^	58.7	<0.001
Walking speed (m/s)	1.34 ± 0.21	1.35 ± 0.20	1.19 ± 0.23 ^N,P^	1.01 ± 0.22 ^N,P,S^	16.1	<0.001

Values are expressed as the mean ± standard deviation. ANOVA, analysis of variance; BMI; body mass index, SMI; skeletal muscle mass index; ^N^ = Difference vs. Normal; ^P^ = Difference vs. Presarcopenia; ^S^ = Difference vs. Sarcopenia; ^D^ = Difference vs. Dynapenia (*p* < 0.05).

**Table 2:** Comparison of gait parameters.

	Normal (*n* = 187)	Presarcopenia (*n* = 79)	Sarcopenia (*n* = 25)	Dynapenia (*n* = 16)	ANCOVA	
					F Value	*p* Value
Walking speed (m/s)	1.34 ± 0.21	1.35 ± 0.20	1.19 ± 0.23 ^N^	1.01 ± 0.22 ^N,P,S^	13.53	<0.001
Cadence (steps/min)	129.05 ± 11.55	129.84 ± 9.88	127.73 ± 11.42	120.34 ± 10.64 ^N,P^	3.16	0.025
Step length (cm)	62.35 ± 7.74	62.37 ± 7.51	56.17 ± 9.19 ^N^	50.15 ± 9.22 ^N,P^	13.55	<0.001
Step width (cm)	7.53 ± 2.79	6.84 ± 2.69	7.04 ± 2.87	9.66 ± 2.84 ^N,P,S^	3.38	0.019
Gait angle (°)	7.04 ± 2.74	6.38 ± 2.68	7.49 ± 3.71	11.31 ± 3.86 ^N,P,S^	10.91	<0.001
Foot angle (°)	2.53 ± 4.46	2.72 ± 5.19	2.27 ± 5.52	1.74 ± 5.14	0.29	0.832
Stance time (s)	0.60 ± 0.07	0.59 ± 0.07	0.63 ± 0.08	0.65 ± 0.10 ^N^	3.39	0.018
Swing time (s)	0.33 ± 0.07	0.33 ± 0.06	0.32 ± 0.06	0.34 ± 0.07	0.37	0.777
Double stance time (s)	0.13 ± 0.05	0.12 ± 0.05	0.15 ± 0.06	0.15 ± 0.06	1.81	0.145

Values are expressed as the mean ± standard deviation. ANCOVA, analysis of covariance. The symbols ^N, P, S, D^ represent a significant between-group difference adjusted with age and body mass index: ^N^ = Difference vs. Normal; ^P^ = Difference vs. Presarcopenia; ^S^ = Difference vs. Sarcopenia; ^D^ = Difference vs. Dynapenia (*p* < 0.05).

## Data Availability

The data used to support the findings of this study are available from the corresponding author upon request. The data are not publicly available because they contain information that can compromise the privacy of research participants.
